# Impact of sodium hexametaphosphate in fluoride toothpaste on initial enamel erosion

**DOI:** 10.1590/1807-3107bor-2026.vol40.083

**Published:** 2026-10-23

**Authors:** Márjully Eduardo Rodrigues da Silva, Alberto Carlos Botazzo Delbem, Alana Semenzin Rodrigues, Priscila Toninatto Alves de Toledo, Denise Pedrini

**Affiliations:** (a) Universidade Estadual Paulista – Unesp, School of Dentistry, Department of Preventive and Restorative, Araçatuba, SP, Brazil.; (b) Universidade Estadual Paulista – Unesp, School of Dentistry, Department of Diagnosis and Surgery, Araçatuba, SP, Brazil.

**Keywords:** Dental Enamel, Fluorides, Phosphates, Tooth Erosion, Dentifrices

## Abstract

This *in vitro* study evaluated the effect of fluoride (F) toothpaste, containing or not sodium hexametaphosphate (HMP), on inhibiting or rehardening initial enamel erosion. Sound (n = 48) and softened (n = 48) bovine enamel blocks were selected by surface hardness (SH) and treated with experimental toothpaste (12 blocks/group): Placebo, 1,100 ppm F, 1% HMP, and 1,100 ppm F + 1% HMP. Enamel blocks were immersed in toothpaste/human saliva slurry once for 2 minutes followed by 4 erosive challenges in citric acid (pH 3.5, 0.75%, 1 minute each, under stirring). The percentage of surface hardness change (%SH) was calculated immediately after demineralization, after treatment and 1^st^, 2^nd^, 3^rd^, and 4^th^ erosive challenge. To assess each toothpaste's ability to form precipitates on enamel, softened enamel blocks (n = 3/group) were treated with the toothpastes and analyzed by SEM/EDX. Data were submitted to 2-way ANOVA followed by the Student-Newman-Keuls test (p < 0.05). The Placebo group showed the lowest capacity to inhibit enamel erosion and reharden softened enamel (p < 0.001). Hardness loss increased during repeated acid challenges for all groups. Toothpaste containing 1,100 ppm F and 1% HMP maintained lower hardness loss than 1,100 ppm F alone after the fourth challenge in sound (−20.8% vs. −31.0%) and softened enamel (−31.3% vs. −39.6%) (p < 0.001). Toothpastes containing HMP produced a more uniform and thicker precipitate layer. Toothpaste containing 1,100 ppm F and 1% HMP demonstrated greater protection against initial erosion and greater rehardening of softened enamel than 1,100 ppm F.

## Introduction

Dental erosion is the chemical loss of mineralized tooth substance caused by exposure to acids not derived from oral bacteria. Erosive tooth wear is tooth wear in which dental erosion is the primary etiological factor; it is increasingly recognized as a common condition in pediatric dentistry, is mostly diagnosed in its initial stages, and increases significantly with age.^1^ Therefore, besides establishing causal preventive approaches, therapeutic measures must be effective,^2^ especially in the early stages of the erosion process. The initial stages (enamel softening) are featured by the dissolution of the outermost enamel layers without loss of its structure.^3^ Although calcium fluoride, fluorapatite, or calcium phosphate deposits formed from fluoride toothpaste can reduce or harden the softened enamel,^3,4^ their dissolution pH is exceeded during the erosive process.^5^ In order to reduce the limitations of fluoride toothpaste,^6,7^ the addition of sodium trimetaphosphate has been shown to possibly increase its anti-erosive potential^8,9^ given that sodium trimetaphosphate blocks acid diffusion by reducing deep enamel softening.^8^

Another phosphate with a strong affinity for hydroxyapatite is sodium hexametaphosphate (HMP), probably due to the presence of six anionic groups in the ring of its molecule.^10,11^ Fluoride toothpaste containing HMP has been shown to reduce mineral enamel loss.^12–15^ This effect may be due to the capacity of HMP to absorb onto the enamel surface and consequently increase calcium and phosphate adsorption and diffusion into the enamel.^10^ *In vitro* ^14^ and *in situ* ^13^ studies have shown that enamel lesion depth was 53% and 25% lower, respectively, when using toothpaste containing 1,100 ppm F and 1% HMP compared to toothpaste containing 1,100 ppm F but no HMP. Even though a solution containing 1% HMP and 1,100 ppm F decreases the hydroxyapatite dissolution rate during repeated acid (pH 3.2) challenges,^14^ this effect needs to be tested in an erosive model utilizing tooth enamel and a toothpaste formulation. In addition, verification of the capacity of F-toothpaste containing HMP to inhibit or harden softened enamel is required.

The aim of this *in vitro* study was to evaluate whether F-toothpaste (1,100 ppm F) containing 1% HMP could inhibit early enamel erosion and reharden softened enamel. The null hypothesis of the study was that toothpaste containing F and HMP would have a similar capacity as F-toothpaste without HMP to inhibit or harden softened enamel.

## Methods

### Toothpaste formulation and fluoride assessment

The toothpastes contained the following components: titanium dioxide, carboxymethyl cellulose, methyl-p-hydroxybenzoate sodium, saccharin, mint oil, glycerin, abrasive silica, sodium lauryl sulfate, and deionized water.^9,13^ HMP (CAS 68915-31-1, Sigma-Aldrich, St. Louis, USA) was added at a concentration of 1% to the toothpaste containing 1,100 ppm F as NaF (Vetec Química Fina Ltda., Duque de Caxias, Brazil).^13,14^ Toothpastes without F or HMP (Placebo) and containing 1,100 ppm F or 1% HMP were also prepared. Total F (TF) and ionic F (IF) concentrations (ppm F, mean [SD]) of all toothpastes were confirmed^9,13^ prior to the beginning of the experiment using a fluoride ion-selective electrode (9409 BN, Thermo Scientific, Beverly, USA): Placebo (18.9 [7.5] and 18.2 [2.7]); 1,100 ppm F (1,373.9 [33.4] and 1,212.1 [69.9]); 1,100 ppm F + 1% HMP (1,342.9 [77.4] and 1,215.5 [37.2); and 1% HMP (20.6 [0.5] and 17.5 [1.3]).

### Preparation of enamel blocks and experimental groups

Enamel blocks (4 × 4 mm) were obtained from bovine incisors and serially polished with water-cooled silicon carbide paper discs (600, 800, and 1,200 grades; Extec, Enfield, USA) to remove ~200 μm from the enamel surface.^8,9^ A sample size of 12 blocks was calculated based on a pilot study, considering the surface hardness as the primary result (KHN), the mean difference between the groups and a standard deviation of 10 KHN and 7 KHN, respectively, an α-error of 0.05, and power (1-β) of 0.80, using the statistical program Sigma Plot version 12.0 (Systat Software Inc., San Jose, USA).

Surface hardness (SH) was determined as follows: a reference indentation was made 1,000 μm from the central region of the enamel block (Knoop diamond, 500 g, 10 seconds, Shimadzu HMV-2000, Shimadzu Corporation, Kyoto, Japan).^3^ Thereafter, five indentations 100 μm apart were made (initial SH: SHi) 200 μm from the vertex of the reference indentation (Knoop diamond, 25 g, 10 seconds, Shimadzu HMV-2000).^3,9,12–14^ Blocks with average SHi between 350.0 and 380.0 KHN,^3,9,12–14^ with flat enamel surfaces and without cracks, scratches, or hypoplasia were selected. All surfaces were covered with nail polish, except the surface to be tested. Enamel blocks (n = 96) were randomly distributed according to the average (confidence interval) SH of the total blocks between the four treatments with experimental toothpastes: Placebo (without F and HMP), 1,100 ppm F, 1,100 ppm F + 1% HMP, and 1% HMP.

### Collection of the human saliva

Paraffin-stimulated saliva was collected in the morning 1 h after breakfast from healthy volunteers of both genders (aged 22–28 years, n = 6), according to the protocol approved by the Research Ethics Committee (CAAE: 03226118.6.0000.5420) following the Declaration of Helsinki. Immediately after collection, saliva samples were pooled and centrifuged for 10 min at 4°C and 2,000×*g*. The supernatants were stored at −72°C into 50 mL bottles.^16^ The pooled saliva contained 24.0 (3.7) μg Ca/mL and 12.3 (0.1) μg P/mL at pH 7.4 (0.2).

### Analysis on sound enamel

Sound enamel blocks (n = 48; 12/group) were immersed (2 mL/block) in a suspension of toothpaste and human saliva (1:3 – wt:wt) once for 2 minutes, and then washed in deionized water for 30 seconds. The acid challenge consisted of individual immersion of each block in 4 mL of citric acid (0.75%, pH 3.5, Synth, Brazil) under agitation (100 rpm) for 1 minute at room temperature (25°C), followed by washing with deionized water for 30s.^3,17^ In total, four erosive challenges of 1 minute each were performed after treatment with the experimental toothpastes. Surface hardness measurements were performed after toothpaste treatment (SHt) to calculate the percentage of surface hardness change (%SH=[(SHt-SHi)/SHi]×100), and after 1^st^, 2^nd^, 3^rd^ and 4^th^ acid challenges (SHc) at 400, 600, 800, 1,000, and 1,200 μm from the right apex of the largest impression (Figure 1A) to calculate the percentage of surface hardness change (%SH=[(SHc-SHi)/(SHi)]×100).^3,17^

**Figure:**
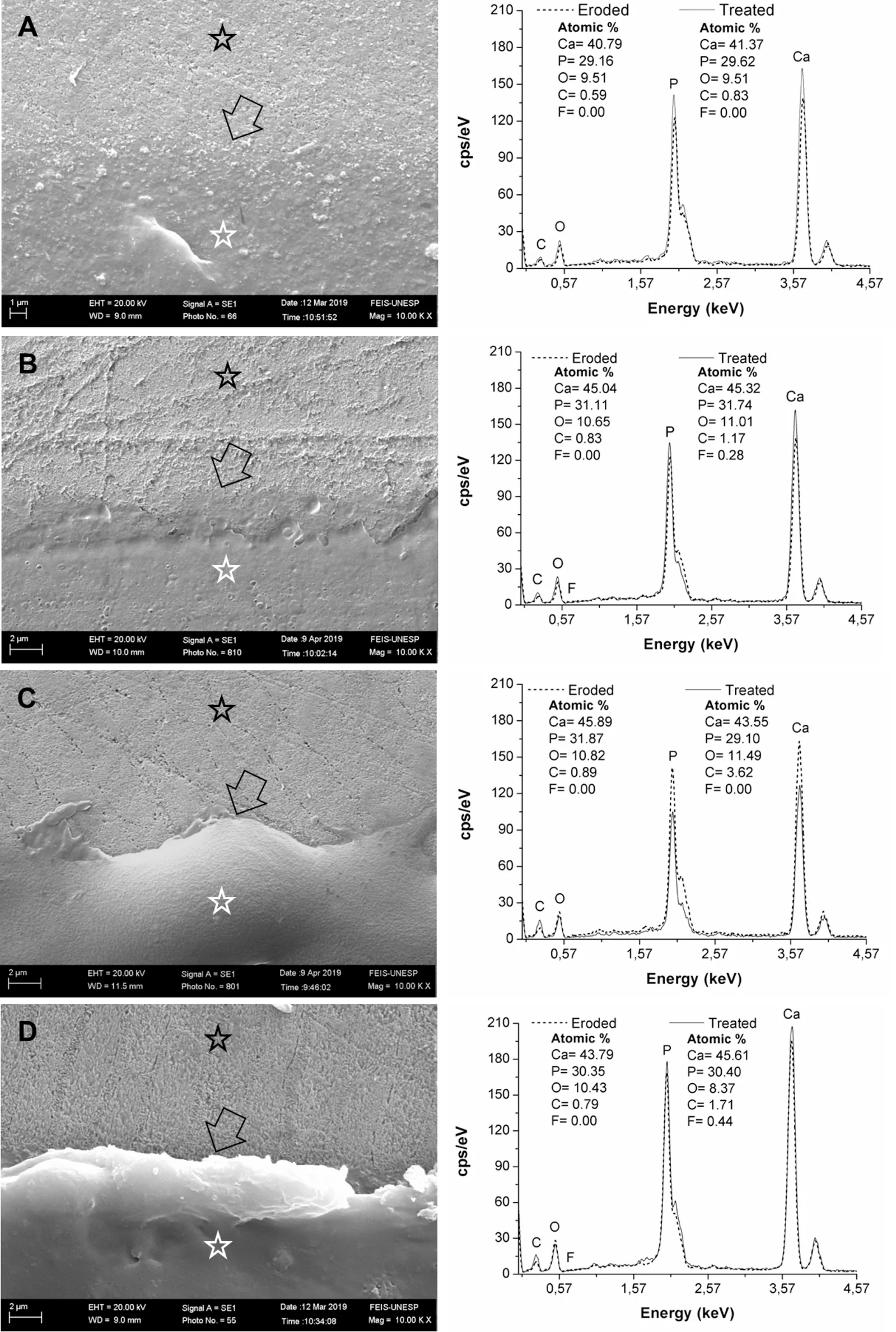
SEM images of a representative specimen in different groups and the respective spectra from EDX analysis: (A) Placebo, (B) 1,100 ppm F, (C) 1% HMP, and (D) 1,100 ppm F + 1% HMP. Black asterisks indicate eroded enamel. White asterisks indicate precipitated enamel formed after the treatments. Arrows indicate the limit between eroded and precipitated enamel formed after the treatments.

### Analysis on softened enamel

Sound enamel blocks were immersed in 4 mL of citric acid (0.75%, pH 3.5, Synth, Brazil) under agitation (100 rpm) for 1 minute at room temperature to produce initial enamel softening,^3,17^ then washed with deionized water for 30 s. After measuring the SH of the softened enamel, the percentage of softening was calculated ([(SHs −SHi)/SHi)]×100). The blocks (n = 48; 12/group) were then treated with the toothpaste/human saliva slurry, washed, and SHt was determined again to calculate the hardening percentage of the softened enamel ([(SHt−SHs)/SHs)]×100).^3^ Next, the blocks were submitted to four acid challenges, as previously described, to verify the residual effect of the treatments. Final SH measurements were performed after each acid challenge to calculate %SH ([(SHc−SHs)/SHs)]×100), as described above.

### Scanning electron microscopy (SEM) and energy-dispersive X-ray (EDX)

In order to verify the capacity of the toothpastes to produce precipitates on the enamel surface, softened enamel blocks (n = 3/group) were prepared as previously described. Half of each surface was then insulated with nail polish and the blocks were treated with toothpaste slurry in human saliva for 2 minutes and washed in deionized water for 30 s.

Thereafter, the nail polish was removed with a scalpel blade and acetone, and the blocks were washed again and dried with absorbent paper. Next, the enamel blocks were mounted on stubs using double-sided carbon tape and taken to a sputter coater (Quorum - Q150T E, West Sussex, UK) to be covered with a gold layer of ~5 nm for 90 s. The images of each sample were obtained by SEM (Carl Zeiss, EVO ILS15, Carl Zeiss NTS LTD, Germany), at 10,000 × magnification and 20 kV, and the presence or absence of precipitate on the enamel surface was evaluated.^3^ The chemical elements in these precipitates were determined utilizing energy-dispersive X-ray spectroscopy (Oxford Instruments, INCAx - act, 133 eV, England) at 20 keV, with ~2 μm of spatial resolution and a count time of 210 s.

### Statistical analysis

Experimental groups (Placebo, 1,100 ppm F, 1% HMP, and 1,100 ppm F + 1% HMP), experimental phases (baseline, posttreatment, and 1^st^, 2^nd^, 3^rd^, and 4^th^ acid challenges), and enamel conditions (sound and softened) were considered as fixed factors. Surface hardness and %SH were used as dependent variables. Data presented normal (Shapiro-Wilk test) and homogeneous (Cochran test) distributions and were thus submitted to two-way repeated-measures analysis of variance followed by the Student-Newman-Keuls test. The analysis was performed with SigmaPlot software (version 12.0, SigmaPlot, Systat Software Incorporation, San Jose, CA, USA), adopting a significance level of 5%.

## Results

From sound enamel (Table), it was clear for all toothpastes that with each erosive challenge there was an increase in surface hardness loss (p < 0.001). However, toothpastes with F and/or HMP led to lower hardness loss than the placebo (p < 0.001). HMP toothpastes produced a greater hardness gain after treatment and lower hardness loss after all erosive challenges than 1,100 ppm F (p < 0.05). The F/HMP association reduced hardness loss at all time points compared to the other toothpastes (p < 0.001).

**Table:** Mean (SD) surface hardness and percentage of surface hardness change (%SH) as a function of toothpastes and experimental phases in sound and demineralized enamel (n = 12).

Toothpastes	Experimental Phases												
	Baseline*		Post-treatment		1^st^ challenge		2^nd^ challenge		3^rd^ challenge		4^th^ challenge		
	Sound**	Softened***	Sound	Softened	Sound	Softened	Sound	Softened	Sound	Softened	Sound	Softened	
Knoop surface hardness													
Placebo	374.8^aA^	335.8^bA^	376.6^aA^	337.7^bA^	329.1^cA^	296.3^dA^	282.6^eA^	252.7^fA^	237.4^gA^	199.1^hA^	190.5^jA^		169.6^kA^
	(1.9)	(3.9)	(1.5)	(4.0)	(3.8)	(4.1)	(7.2)	(1.9)	(4.0)	(3.7)	(6.3)		(4.5)
1,100 ppm F	375.0^aA^	334.6^bA^	383.0^cB^	347.8^dB^	349.5^dB^	314.1^eB^	327.3^fB^	291.7^gB^	313.1^hB^	237.3^iB^	258.4^jB^		202.1^kB^
	(2.3)	(2.4)	(2.9)	(2.5)	(2.2)	(2.8)	(3.4)	(6.0)	(3.2)	(5.0)	(4.4)		(2.9)
1% HMP	374.5^aA^	333.5^bA^	408.7^cC^	352.7^dC^	381.2^eC^	302.7^fC^	356.7^gC^	250.0^hA^	332.5^iC^	219.1^jC^	274.8^kC^		207.0^lC^
	(4.1)	(6.0)	(5.3)	(4.6)	(4.4)	(5.3)	(5.2)	(5.3)	(5.7)	(4.6)	(6.3)		(3.0)
1,100 ppm F/	375.5^aA^	334.4^bA^	418.5^cD^	366.2^dD^	402.6^eD^	321.9^fD^	380.7^gD^	289.1^hB^	360.5^dD^	243.4^iD^	297.4^jD^		229.8^kD^
1% HMP	(1.8)	(2.3)	(4.3)	(2.2)	(3.2)	(6.7)	(3.4)	(5.1)	(6.8)	(6.3)	(3.5)		(4.2)
Percentage of surface hardness change													
Placebo	0^aA^	−10.4^bA^	0.5^aA^	0.6^aA^	−12.2^cA^	−11.8^cA^	−24.6^dA^	−24.7^dA^	−36.7^eA^	−40.7^fA^	−49.2^gA^		−49.5^gA^
		(0.8)	(0.4)	(0.1)	(1.1)	(0.6)	(2.0)	(0.9)	(1.1)	(1.7)	(1.7)		(1.0)
1,100 ppm F	0^aA^	−10.8^bA^	2.3^cB^	3.9^dB^	−6.7^eB^	−6.1^eB^	−12.6^fB^	−12.8^fB^	−16.4^gB^	−29.1^hB^	−31.0^iB^		−39.6^jB^
		(0.4)	(1.0)	(0.4)	(0.8)	(0.6)	(1.1)	(1.7)	(0.9)	(1.4)	(1.2)		(0.8)
1% HMP	0^aA^	−10.9^bA^	9.1^cC^	5.8^dC^	1.8^eC^	−9.3^fC^	−4.7^gC^	−25.0^hA^	−11.2^bC^	−34.3^iC^	−26.6^hC^		−37.9^jC^
		(1.7)	(1.1)	(1.0)	(1.3)	(1.1)	(1.7)	(0.7)	(0.7)	(0.9)	(1.4)		(1.5)
1,100 ppm F/	0^aA^	−10.9^bA^	11.5^cD^	9.5^dD^	7.2^eD^	−3.7^fD^	1.4^gD^	−13.5^hB^	−4.0^fD^	−27.2^iD^	−20.8^jD^		−31.3^kD^
1% HMP		(0.8)	(0.8)	(0.6)	(0.8)	(2.0)	(0.8)	(1.6)	(1.6)	(1.8)	(0.9)		(1.1)

Lowercase letters indicate a significant difference between conditions for each toothpaste. Capital letters indicate no significant difference between toothpaste means for each condition (two-way repeated-measures ANOVA, Student-Newman-Keuls, p < 0.05).

* Baseline: surface hardness values before treatments of the sound (SHi) and softened (SHs) enamel blocks, and respective %SH values.

** Sound: sound bovine enamel blocks.

*** Softened: previously softened bovine enamel blocks.

After the initial softening of the enamel, the loss of surface hardness demonstrated no differences among groups (p < 0.05). Following toothpaste treatment, rehardening and a gain in surface hardness were observed for all groups, but with differences between them (p < 0.001), with a higher value for the 1,100 ppm F + 1% HMP group (Table). After the four acidic challenges, hardness loss increased for all groups (p < 0.05), but was lower in the 1,100 ppm F + 1% HMP group (p < 0.001).

Distinct precipitates were evident by SEM on the treated demineralized enamel surfaces from the different toothpastes, forming a thick layer on the enamel surface (Figure). In contrast, exposed apatite crystals and fish-scale "keyholes" or honeycomb structures appeared on the untreated demineralized surface. In this study, the chemical elements identified and considered relevant (> 0.2%) were calcium (Ca), phosphorus (P), carbon (C), oxygen (O), and F. For Placebo and 1,100 ppm F, the precipitate produced a thinner, more homogeneous layer with fluoride toothpaste (Figures A and B), but 1,100 ppm F presented higher peak intensities for Ca, P, C, and F than the untreated demineralized surface (Figure B). A thicker, homogeneous layer of precipitate was observed with toothpastes containing HMP (Figures C and D) with greater amount of C than in the untreated area. The highest intensities of Ca, P, and F were observed for the treated area with toothpaste containing 1,100 ppm F and HMP.

## Discussion

Although the 1,100 ppm F/1% HMP toothpaste formulation induce deep enamel remineralization,^12–14^ it has not yet been demonstrated whether its effect withstands subsequent erosive challenges on sound and softened enamel. The results showed that the F/HMP toothpaste caused the least enamel softening and had the highest ability to reharden softened enamel compared to F-toothpaste without HMP. More specifically, compared with 1,100 ppm F toothpaste, the 1,100 ppm F/1% HMP formulation reduced hardness loss after the fourth erosive challenge by approximately 33% in sound enamel and 21% in previously softened enamel, while producing an approximately 2.4-fold greater post-treatment hardening effect in softened enamel. This indicates that the protective effect of the F/HMP toothpaste persisted during subsequent acid challenges in previously softened enamel. Thus, the null hypothesis was rejected. Furthermore, toothpastes containing HMP produced large quantities of precipitate on the demineralized enamel surface.

In the present study, the dentifrice containing HMP and F (1,100 ppm F) had a greater protective and rehardening effect against enamel demineralization (Table). Although prior studies have shown a limited or no effect of F toothpaste (> 1,000 ppm F) against enamel wear,^6,9,18–20^ in an initial erosive process that produces softened enamel without loss of structure, the 1,100 ppm F demonstrated a significantly higher protective effect compared with placebo. The effect of F toothpaste is known to result from the precipitation of calcium fluoride (CaF_2_-like) on enamel.^6,21^ Furthermore, higher deposition of CaF_2_ occurs on demineralized enamel due to the great availability of calcium and phosphate and large reactivity area.^22,23^ This corroborates the 30% higher mineral gain in demineralized enamel ([SHt−SHs]/[SHi-SHs]*100; calculation not described in Table) compared to sound enamel when treated with a toothpaste containing 1,100 ppm F (Table). Moreover, these deposits presented greater resistance to erosive challenges than sound enamel since %SH showed no difference at the 1^st^ and 2^nd^ erosive challenges. It is important to note that the hardness of the previously softened groups was always lower than that of the sound groups. In contrast, the precipitate induced by 1% HMP toothpaste produced a greater post-treatment surface hardness gain than 1,100 ppm F, corresponding to approximately 9.1% versus 2.1% in sound enamel and 5.8% versus 3.9% in softened enamel, although this effect was less durable over time in softened enamel.

Unlike the calcium phosphate deposits formed on the placebo specimens (Figure A), the presence of HMP makes this layer less susceptible to acids. HMP can adsorb onto sound enamel,^10^ creating a protective layer that hinders acid diffusion into the enamel.^13,14^ The HMP layer presents larger quantities of electron-donor sites that enhance the adsorption of ionic species such as Ca^2+^, H_2_PO_4_^–^, and CaH_2_PO_4_^+^ on enamel.^8,10^ To confirm this ability, the additional experiment showed a massive deposition of the HMP-Ca-PO_4_ layer on demineralized enamel with the HMP toothpastes (Figures C and D), with Ca/P ratio of 1.50, despite a higher amount of C and O, which may indicate a greater presence of carbonates in the precipitate. The greater presence of carbonate and the absence of fluoride in the deposits from the 1% HMP group (Figure) may explain their lower resistance to subsequent acid challenges (Table) compared to the 1,100 ppm F and 1,100 ppm F/1% HMP groups. Unlike treatment with 1,100 ppm F, previously demineralized enamel showed less mineral gain than sound enamel when treated with dentifrices containing 1% HMP. Despite having a more reactive surface, previously demineralized enamel makes more Ca^2+^ and PO_4_^3-^ available to be easily sequestered by a highly chelating agent such as HMP.^15^ Thus, it was established that the effect of HMP is dependent on its concentration in the formulation, being effective at concentrations equal to or less than 1%.^12,15^ As HMP provides a layer that reduces the acid diffusion into the enamel,^10,13,14,24^ previously softened enamel does not seem to favor HMP adsorption since in sound enamel the loss of hardness was smaller (Table). However, the presence of fluoride (Figure D) in the HMP layer minimizes this lack of resistance to acidic challenges.

Contrary to previous studies that used caries models,^12,13^ and based on the data discussed above, the results of the present study demonstrated that the F/HMP association has an additive effect on the erosive process. Although studies have shown that cyclic phosphates compete with fluoride adsorption on enamel,^14,25^ the presence of F induces an increase in HMP adsorption on sound enamel and, consequently, a greater number of calcium phosphate nucleating sites. An adequate F/HMP ratio is necessary to obtain the maximum effect. HMP concentrations above 1% do not improve the effect against demineralization.^12,14,15^ It is probable that the structural arrangement of the HMP layer formed in the presence of fluoride is different from that of HMP alone. The EDX spectrum showed greater intensities of Ca, P, and F in the precipitates formed after treatment with 1,100 ppm F/1% HMP toothpaste (Figure D). The SEM/EDX findings, together with previous hypotheses,^12,13^ suggest that the precipitate formed after treatment with 1,100 ppm F/1% HMP is consistent with an HMP-Ca-F-rich surface layer; however, its exact chemical composition was not directly confirmed in the present study. This precipitate layer may have contributed to greater retention and deposition of calcium, phosphate, and fluoride on the enamel surface, helping to explain the higher rehardening ability of the F/HMP formulation compared with the 1,100 ppm F toothpaste. Furthermore, the inhibitory effect persisted during repeated acid challenges as observed in a previous study utilizing hydroxyapatite.^14^

Regardless of enamel condition, F/HMP-containing toothpaste provided greater protection against initial enamel softening and a greater ability to reharden previously softened enamel under the conditions of this study. From a clinical perspective, these findings may be particularly relevant for patients at higher risk of erosive tooth wear, such as individuals with frequent acidic beverage consumption, gastroesophageal reflux disease, eating disorders associated with intrinsic acid exposure, or reduced salivary protection, including xerostomia.^1,2,22,23^ The addition of HMP to a conventional fluoride toothpaste may therefore represent a promising strategy to enhance the prevention of early erosive lesions while maintaining a standard fluoride concentration.^13,14^

Nevertheless, these implications should be interpreted with caution because the present *in vitro* model does not fully reproduce the complexity of the oral environment. The experiment was performed using bovine enamel, a single toothpaste exposure under standardized slurry conditions, and repeated citric acid challenges. Although human saliva was used to prepare the toothpaste slurry, the model did not reproduce acquired pellicle maturation, salivary flow and clearance, buffering dynamics, biofilm formation, patient compliance, or the abrasive effect of toothbrushing.^17,19,20,22,23^ Therefore, the present findings should be considered experimental evidence supporting the potential of HMP-containing fluoride toothpaste rather than a direct clinical recommendation. Further *in situ* and clinical studies are needed to determine whether this protective effect is maintained under oral conditions and whether it effectively protects enamel against erosive tooth wear in high-risk patients.^13,15,20^

## Conclusion

Within the limitations of this *in vitro* study, 1,100 ppm F toothpaste containing 1% HMP showed a greater effect than 1,100 ppm F toothpaste alone in inhibiting initial erosion in sound enamel, rehardening previously softened enamel, and improving resistance to repeated erosive challenges.

## Data Availability

The datasets generated during and/or analyzed during the current study are available from the corresponding author on reasonable request.
